# Fat Mass and Obesity-Associated Protein Regulates Granulosa Cell Aging by Targeting Matrix Metalloproteinase-2 Gene Via an N6-Methyladenosine-YT521-B Homology Domain Family Member 2-Dependent Pathway in Aged Mice

**DOI:** 10.1007/s43032-024-01632-6

**Published:** 2024-07-12

**Authors:** Linshuang Li, Le Yang, Lin Shen, Yiqing Zhao, Lan Wang, Hanwang Zhang

**Affiliations:** Reproductive Medicine Center, Tongji Hospital, Tongji Medicine College, Huazhong University of Science and Technology, Jiefang Avenue 1095#, Wuhan, 430030 People’s Republic of China

**Keywords:** Ovarian aging, Granulosa cells, m6A, FTO, YTHDF2, MMP2

## Abstract

In this study, we aimed to investigate the molecular mechanisms of RNA N6-methyladenosine (m6A) modification and how its associated proteins affect granulosa cell aging. A granulosa cell senescence model was constructed to detect the differences in total RNA m6A modification levels and the expression of related enzymes. Changes in downstream molecular expression and the effects on the cellular senescence phenotype were explored by repeatedly knocking down and overexpressing the key genes fat mass and obesity-associated protein** (***FTO*), YT521-B homology domain family member 2 (*YTHDF2*), and matrix metalloproteinase-2 (*MMP2*). There was an increased total RNA m6A modification and decreased expression of the demethylase FTO and target gene *MMP2* in senescent granulosa cells. FTO and MMP2 knockdown promoted granulosa cell senescence, whereas FTO and MMP2 overexpression retarded it. YTHDF2 and FTO can bind to the messenger RNA of MMP2. The extracellular signal-regulated kinase (ERK) pathway, which is downstream of MMP2, retarded the process of granulosa cell senescence through ERK activators. In granulosa cells, FTO can regulate the expression of MMP2 in an m6A-YTHDF2-dependent manner, influencing the activation status of the ERK pathway and contributing to the aging process of granulosa cells.

## Introduction

With the adjustment of population structure in modern society and the postponement of women's first childbearing age, problems associated with ovarian aging have become increasingly severe [1]. A critical aspect of ovarian aging is the decline in the quantity and quality of oocytes. Oocyte development within follicles depends on communication with granulosa cells, which provide nutrients to the oocyte and are vital in maintaining its stability and initiating meiosis [2]. With advancing age, granulosa cells undergo structural degeneration of DNA, proteins, and lipids, negatively impacting the developmental capacity of oocytes [3, 4]. Therefore, it is imperative to investigate the aging process of granulosa cells and devise strategies for addressing ovarian aging.

RNA N6-methyladenosine (M6A) is the most common internal modification in RNA [5]. It plays an important role in a variety of physiological and pathological processes, regulating post-transcriptional modifications such as translation, processing, stability, splicing and degradation of target RNA. [6, 7]. The key components involved in the dynamic modification of RNA m6A in cells include a demethylase (fat mass and obesity-associated protein [FTO]), methylase [8], and methylation recognition enzyme (YT521-B homology domain family member 2 [YTHDF2]) [9]. FTO modulates gene expression and pathophysiological processes by controlling RNA metabolism, particularly stability [10], by reducing the m6A modification levels in the target RNA. Conversely [11], YTHDF2 degrades the messenger RNA (mRNA) of target genes with m6A modifications [12], influencing processes such as cell proliferation, apoptosis, oocyte competence, and early zygotic development [13]. However, the specific effects of FTO and YTHDF2 on granulosa cell senescence through m6A modification remain unclear, highlighting the need for further investigation.

Matrix metalloproteinase 2 (MMP2), a member of the matrix metalloproteinase family, is crucial in follicle formation, ovulation, and corpus luteum formation in the ovary; it modifies specific components of the extracellular matrix [14, 15]. The proteolytic activity of matrix metalloproteinases is vital in various physiological processes such as tissue remodeling, morphogenesis, embryogenesis, angiogenesis, organ senescence, apoptosis, and wound healing [16, 17]. Notably, some studies have reported reduced MMP2 expression in the ovaries of older women [18]. However, there is no research linking the involvement of MMP2 in ovarian physiology or pathological aging to RNA m6A methylation.

This study aimed to investigate the impact of RNA m6A modification-related enzymes on granulosa cell senescence and the potential mechanism of MMP2 regulation. Our findings suggest that the decline in FTO and MMP2 expression, as well as the diminished activation of the ERK pathway, are correlated with the progression of senescent granulosa cells. We propose that FTO may play a role in regulating granulosa cell senescence through an m6A-YTHDF2-dependent mechanism, with granulosa cells potentially influencing ovarian aging by interacting with oocytes. Overall, these findings offer a novel avenue for a deeper understanding of ovarian aging.

## Materials and Methods

### Ethics and Animals

The study involved 6-week-old and 9-month-old Institute of Cancer Research female mice obtained from the Beijing Spafford Company (Beijing, China). All experimental procedures were conducted following the regulations of the Ethics Committee of Tongji Hospital (TJ-IRB20210213). Following euthanasia through cervical dislocation, the ovaries were extracted, the connective tissue around the ovary was carefully removed under a stereomicroscope, one side of the ovary was preserved in a tissue fixative, and the other side was stored at -80 ℃ for future use.

## Cell Culture and Treatment

The human granulosa-like tumor (KGN) cell line was obtained from Wuhan Procell Life Science & Technology Co., Ltd. (Wuhan, China) and was originally sourced from the ATCC Cell Bank (American Type Culture Collection, Manassas, VA, USA). The cells were cultured in a full medium of DMEM/F12 (Procell) supplemented with 10% fetal bovine serum (FBS) (Gibco, Billings, MT, USA) for growth and expansion in an incubator set at 37 °C with 5% CO_2_. The cell line was authenticated using a tandem repeat pattern and was routinely screened for mycoplasma contamination. To establish a granulosa cell senescence model, KGN cells were cultivated for two weeks in a full medium of DMEM/F12 containing 20 mg/mL D-( +)-galactose.

## Western Blot

Ovarian tissue and cell samples were processed by adding them to a radioimmunoprecipitation assay buffer (Servicebio, Wuhan, China), along with a protease and phosphatase inhibitor mixture (Servicebio) and grinding in a cryogenic tissue grinder. The resulting lysate was incubated on ice and then centrifuged. Protein concentration was determined using the BCA protein assay kit (Beyotime, Beijing, China), after which the samples were denatured by heating in boiling water for 10 min. Following this, the proteins underwent separation utilizing 10% SDS-gel electrophoresis (Yesen, Dongguan, China) and were moved to a PVDF membrane (Millipore, Burlington, MA, USA). Next, the membrane was blocked with 5% skim milk (Servicebio) and 0.1% Tween-20 (Servicebio) at ambient temperature for 1 h, then proceeded to overnight incubation with diluted primary antibodies at 4 °C. Antibodies against FTO (Proteintech, Rosemont, IL, USA), YTHDF2 (Proteintech), MMP2 (Proteintech), BCL-2 (Abcam, Cambridge, UK), BAX (ABclonal, Woburn, MA, USA), ERK (CST, USA), and p-ERK (CST) were employed in the study. According to the instructions of the manufacturer of the reagents, different primary antibodies are diluted at different concentrations, FTO, YTHDF2, MMP2, BAX are diluted at 1:1000, while BCL-2, ERK, p-ERK are diluted at 1:2000. Following the washing step, the membranes underwent incubation with secondary antibodies (Servicebio) with the dilution of 1:2000, visualized using electrochemiluminescence (Servicebio), and quantified using ImageJ software (NIH, Bethesda, MD, USA).

## Real-Time Quantitative PCR (RT-qPCR)

Granulosa cells were subjected to total RNA extraction using the RNA-easy Isolation Reagent (Vazyme, Nanjing, China), followed by RNA quality assessment. According to the manufacturer's guidelines, reverse transcription was carried out using the Hifair III 1st strand complementary DNA synthesis SuperMix for a quantitative polymerase chain reaction (qPCR) kit (Yesen). PCR amplification was performed using Hieff qPCR SYBR Green Master Mix kit (Yesen). Program design and refinement were performed in accordance with the manufacturer instructions. Each sample was tested in duplicate using GAPDH as an endogenous control. Genes such as P16, p21, and p27 are associated with cell cycle regulation, and senescent cells typically exhibit cell cycle arrest [19]. Senescence-related genes like IL-1α, IL-1β, IL-6, IL-8, and CCL2 are linked to secretory phenotypes [20]. The primers utilized for qPCR can be found in Table 1.

**Table 1:** Reference genes for gene expression normalization

Gene name	NCBI gene ID	NM-number	Forward primer sequence (5’- > 3’)	Reverse primer sequence (5’- > 3’)	Product length (bp)	Tm-value (Forward, °C)	Tm-value (Reverse, °C)
*P16*	1029	NM_001195132.2	GAGGGCTTCCTGGACACG	TCTATGCGGGCATGGTTA	176	59.73	55.62
*P21*	1026	NM_001374511.1	GGGATGAGTTGGGAGGAG	AAGGGTACAAGACAGTGACAGG	101	55.94	59.63
*IL-la*	3552	NM_000575.5	ATTTGACATGGGTGCTTAT	TACCTGTGATGGTTTTGGG	155	52.05	54.65
*IL-1β*	3553	NM_000576.3	GATGGCTTATTACAGTGGC	TAGTGGTGGTCGGAGATT	138	53.52	54.37
*IL-6*	3576	NM_001371096.1	AGCCCTGAGAAAGGAGACA	CCAAAAGACCAGTGATGAT	167	52.63	42.11
*IL-8*	6347	NM_001354840.3	ATAAAGACATACTCCAAACCT	ACCTTCTCCACAACCCTC	167	51.82	53.61
*CCL2*	4313	NM_002982.4	TAGCAGCCACCTTCATTC	CTTGGGGTCAGCACAGAT	200	54.23	56.52
*MMP2*	79,063	NM_001302510.2	GTTCATTTGGCGGACTGT	AGGGTGCTGCTGAGTAG	171	55.5	58.28
*FTO*	2597	NM_001080432.3	CTCATCTCGAAGGCAGGGGAT	GTGAAGGGGTATCGCCAAAC	92	58.95	58.91
*GAPDH*	51,441	NM_001357943.2	AGAAGGCTGGGGCTCATTTG	AGGGGCCATCCACAGTCTTC	258	60.32	61.57
*YTHDF2*		NM_001173128.2	ATGTGAGGTGGATTTTGT	CACTGGTTTATTCTCGTTG	83	52.22	51.55

## RNA Binding Protein Immunoprecipitation

We performed RNA immunoprecipitation (RIP) experiments in KGN cells to investigate the possibility that MMP2 directly binds FTO and YTHDF2. Radioimmunoprecipitation assays were performed using the Magna RIP RNA-binding protein immunoprecipitation kit (Millipore) and specific antibodies against FTO (Proteintech) and YTHDF2 (Proteintech) according to the manufacturer’s instructions. The binding of FTO and YTHDF2 to mRNA transcripts was then assessed using qPCR.

## Lentiviral Infection and Small Interfering RNA (siRNA) Transfection

Cells were planted in a 24-well dish and grown until they achieved 70% confluence, after which the cell culture medium was removed, and lentivirus obtained from Shanghai Genechem Company (Shanghai, China) was used to infect KGN cells following the manufacturer’s instructions. Puromycin (Cayman Chemical, Ann Arbor, MI, USA) was used to screen the infected cells to establish stable transgenic strains. The manufacturer's instructions were followed for transfecting cells with siRNA, which was obtained from Guangzhou Ribo Company (Guangzhou, China). This involves adding siRNA, serum-free DMEM/F12 medium, and transfection reagent (Ribo) to each well. After 6 h of starvation, remove the transfection solution, add complete DMEM/F12 medium containing 10% fetal calf serum, and continue culturing for 24–48 h.

## Immunofluorescence Staining

Cells were seeded in a 24-well plate, followed by removal of the culture medium, rinsing, and fixation with 4% paraformaldehyde (Servicebio). Subsequently, the membrane was made permeable using 0.5% TritonX-100 (Servicebio), blocked with 1% BSA (Servicebio) in PBS, and subsequently exposed to the primary antibody solution as directed, followed by an overnight incubation at 4 °C. Following the washing step, a pre-made fluorescent secondary antibody (Servicebio) was introduced to all wells and left to incubate at room temperature in the absence of light. It is important to mention that DAPI (Servicebio) was applied for nuclear staining, and subsequent visualization and recording of images took place using a fluorescence microscope.

## β-galactosidase Staining

Β-galactosidase staining is commonly used to assess cellular senescence the increased level of β-galactosidase activity, as a prominent marker of high lysosomal activity and lysosomal content, was observed in both senescence and quiescence status but clearly higher in senescence [21]. Cells were added to a 6-well plate, followed by removal of the cell culture medium and subsequent washing of the cells. The protocol of the β-galactosidase Staining Kit (Beyotime) was carried out according to the provided guidelines. Subsequently, images were examined and captured utilizing a standard light microscope.

## Terminal Deoxynucleotidyl Transferase dUTP Nick End Labeling (TUNEL) Assay

Cells were seeded in a 24-well plate, the cell culture medium was discarded, and the cells were washed and fixed with 4% paraformaldehyde (Servicebio). Following this step, the membrane underwent permeabilization using 1 × PBS with 0.3% Triton X-100 (Servicebio). After washing, the cells were exposed to the TUNEL reagent (Beyotime) to create the TUNEL staining solution according to the provided guidelines. Subsequently, the labeled cells were examined, and pictures were taken utilizing a fluorescence microscope.

## Reactive Oxygen Species (ROS) Assay

The KGN cells were placed into a 24-well plate, and the growth medium was taken out. To achieve a concentration of 10 μmol/L, Dichlorodihydrofluorescein diacetate was diluted based on the guidelines provided in the Reactive Oxygen Species Assay Kit (Beyotime). The KGN cells were then treated with the diluted solution and placed in a cell culture chamber at 37 °C for 20 min. To promote adequate interaction between cells and probes, rotate the plate every 3–5 min during incubation. After incubation, cells were washed, and images were taken using a fluorescence microscope.

## Assessment of Mitochondrial Membrane Potential (MMP)

Following the guidelines provided by the manufacturer, the cells were placed into a 24-well plate for the MMP assay kit containing JC-1 (Beyotime). Once taken out of the incubator, the medium was removed, cells were washed, and the JC-1 staining solution was applied. The plate was then returned to the cell culture incubator and incubated at 37 °C for 20 min. After the incubation period, the supernatant was removed, cells were washed, and images were captured using a fluorescence microscope.

## Statistical Analysis

All experimental results were independently replicated at least three times to ensure the reliability and reproducibility of the findings. Statistical analysis was performed using GraphPad Prism 8 software from GraphPad Software in Boston, MA, USA. The study utilized a completely randomized design with two independent samples and a small sample size, data collected consisted of measures that were assessed using SPSS and were confirmed to follow a normal distribution. Results are reported as the mean ± standard error, with a two-tailed Student's t-test used for pairwise group comparisons and analysis of variance for comparisons among multiple groups. A significance level of P < 0.05 was employed to determine statistical significance in the study.

## Results

### Increased Levels of m6A Modifications in Senescent Granulosa Cells

**Fig. 1:**
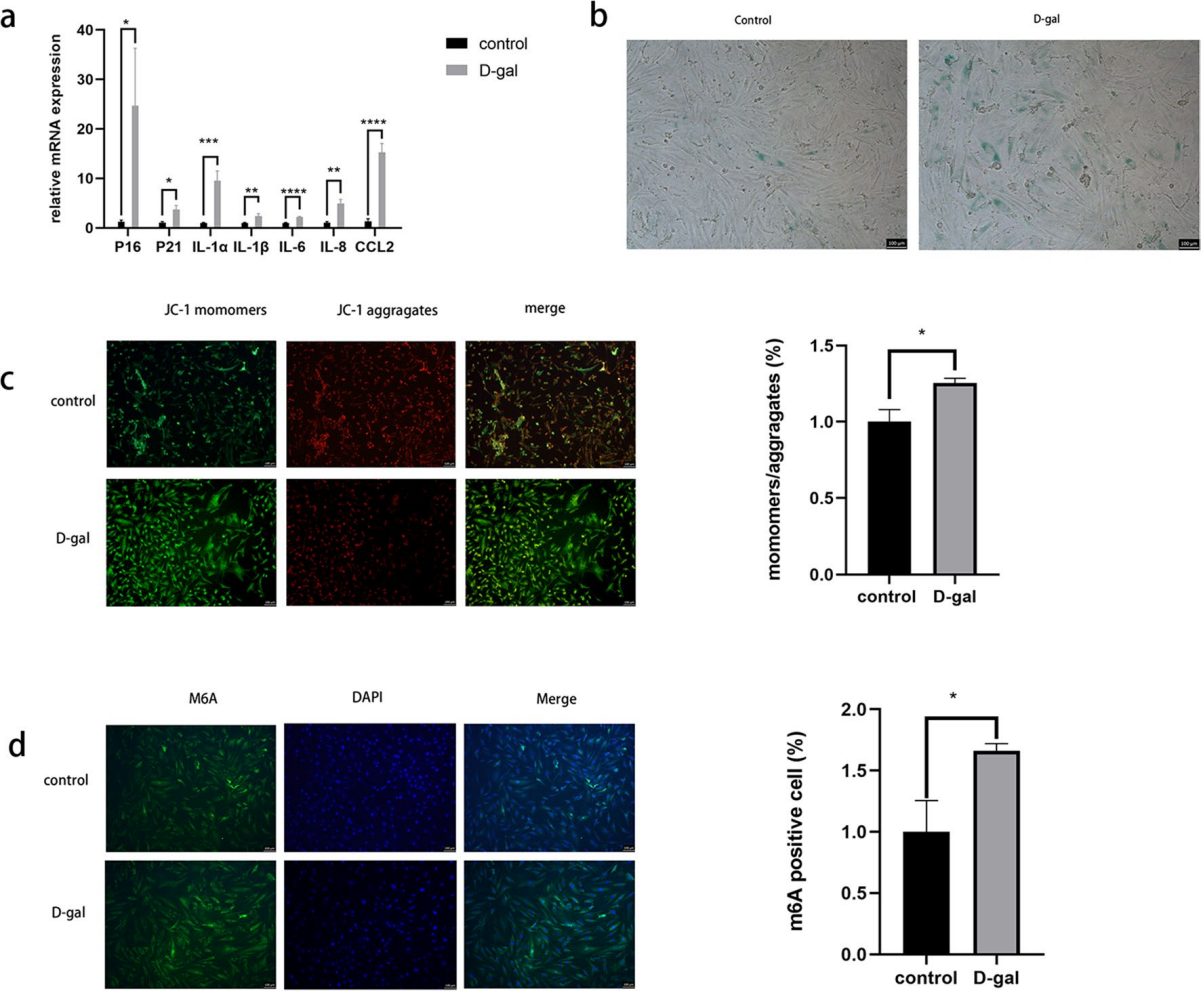
Increased levels of m6A modifications in senescent granulosa cells. (**a**) RT‐qPCR revealed the transcript levels of these genes after D-( +)-Galactose treatment; (**b**) the β-galactosidase staining detected senescent cells in the control and D-( +)-Galactose groups; (**c**) intracellular MMP was measured using JC-1 staining in the control and D-( +)-Galactose groups; (**d**) Immunofluorescence results showed RNA m6A modification level after D-( +)-Galactose treatment. *P < 0.05; **P < 0.01; ***P < 0.001; ****P < 0.0001. RT-qPCR, quantitative reverse transcriptase polymerase chain reaction; MMP, mitochondrial membrane potential

Previous reports have shown that m6A levels are elevated in the ovaries of aged mice [19]. However, it remains unclear whether increased levels of m6A modification also occur in senescent granulosa cells. Therefore, the cells were treated with complete DMEM/F12 containing 20 mg/mL D-( +)-galactose for 2 weeks to test this hypothesis. P16 and P21 are key components of senescent cells, whereas interleukin (*IL)-1α*, *IL-1β, IL-6, IL-8,* and chemokine (CC-motif) ligand 2 (CCL2) are genes associated with Senescence-Associated Secretory Phenotype. RT-qPCR analysis revealed an increase in the transcription levels of these genes following D-( +)-galactose treatment (Fig. 1a). The proportion of β-galactosidase-stained cells was significantly higher in the treated KGN cells compared with control cells (Fig. 1b). Additionally, the MMP in the D-( +)-galactose group decreased (Fig. 1c). Collectively, these results suggest that senescence was successfully induced in KGN cells. Subsequent immunofluorescence experiments confirmed the presence of elevated m6A modifications in senescent granulosa cells (Fig. 1d).

## Knockdown of FTO Promotes Senescence *in Granulosa* Cells

**Fig. 2:**
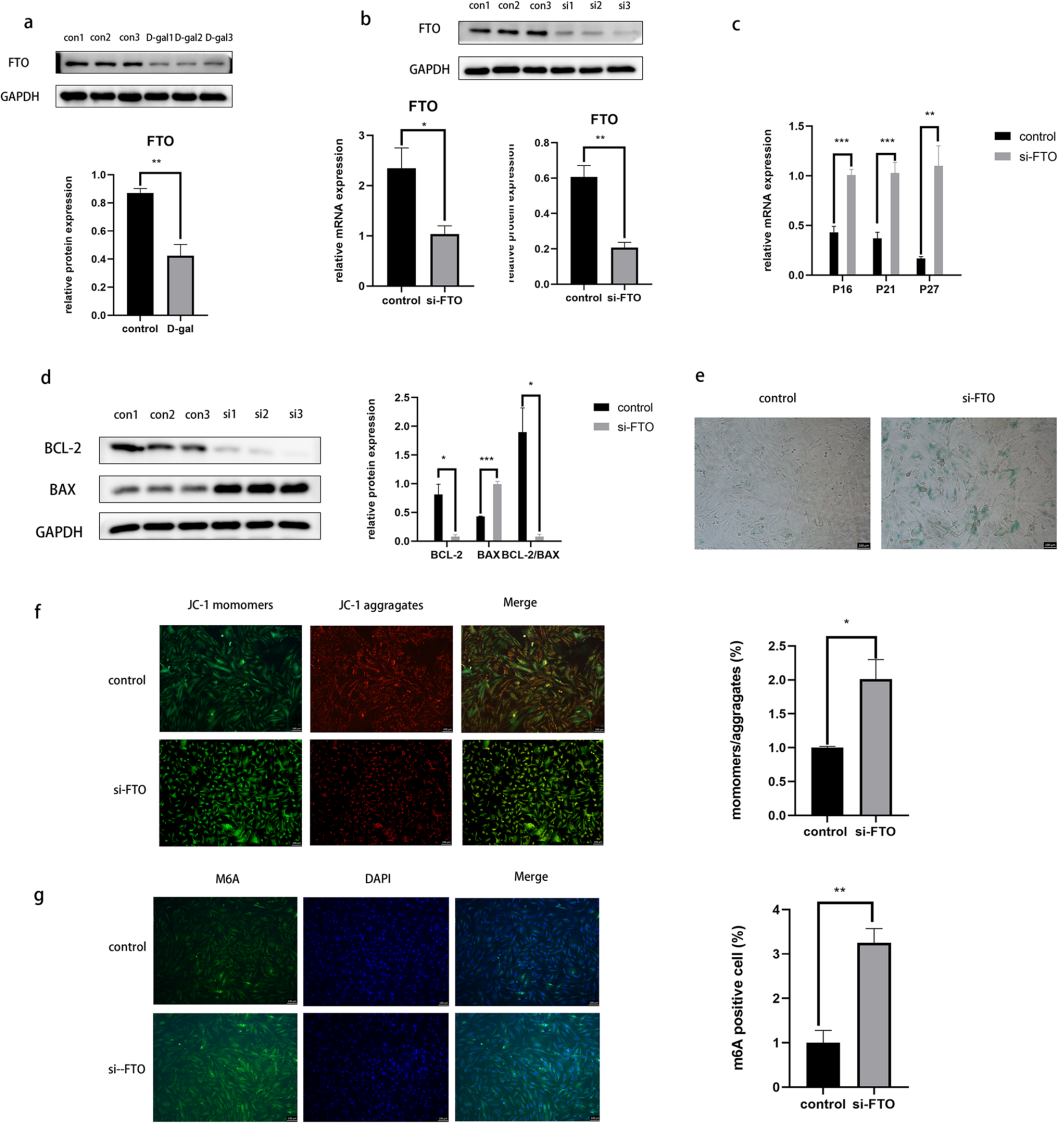
Knockdown of FTO promotes senescence in granulosa cells. (**a**) Western blot results revealed protein levels of FTO after D-( +)-Galactose treatment; (**b**) FTO expression level after transfecting with si-FTO; (**c**) qPCR results revealed the mRNA levels of P16, P21, and P27 after FTO downregulated; (**d**) Western blot results indicated the protein levels of Bax and Bcl‐2 after FTO downregulated; (**e**) the β-galactosidase staining detected senescent cells in the control and FTO downregulated groups; (**f**) intracellular MMP was measured using JC-1 staining in the control and FTO downregulated groups; (**g**) Immunofluorescence results showed RNA m6A modification level after FTO downregulated. *P < 0.05; **P < 0.01; ***P < 0.001. FTO, fat mass and obesity-associated protein; qPCR, quantitative polymerase chain reaction; MMP, mitochondrial membrane potential

Previous experiments have revealed a reduction in the expression of FTO in the ovaries of aged mice [22], prompting us to investigate whether a similar trend occurs in senescent granulosa cells. Western blot analysis confirmed a decrease in FTO expression in the senescent cell group compared with that in the control group (Fig. 2a). To further understand the impact of FTO on senescence in granulosa cells, we conducted loss-of-function assays using siRNA to achieve a minimum of 50% knockdown of endogenous FTO protein and RNA in KGN cells (Fig. 2b). Subsequent qPCR analysis demonstrated the upregulation of P16, P21, and P27 expression in KGN cells compared with control cells (Fig. 2c). Additionally, Western blot results showed a significant increase in Bax expression, a decrease in Bcl-2 expression, and a reduction in the BCL-2/BAX ratio following siRNA transfection (Fig. 2d). Moreover, an increased proportion of β-galactosidase staining-positive cells (Fig. 2e), decreased MMP (Fig. 2f), and elevated RNA m6A modification levels (Fig. 2g) were observed. These findings indicated that the loss of FTO contributed to the promotion of senescence in granulosa cells.

## Overexpression of FTO Inhibits Granulosa Cell Senescence

**Fig. 3:**
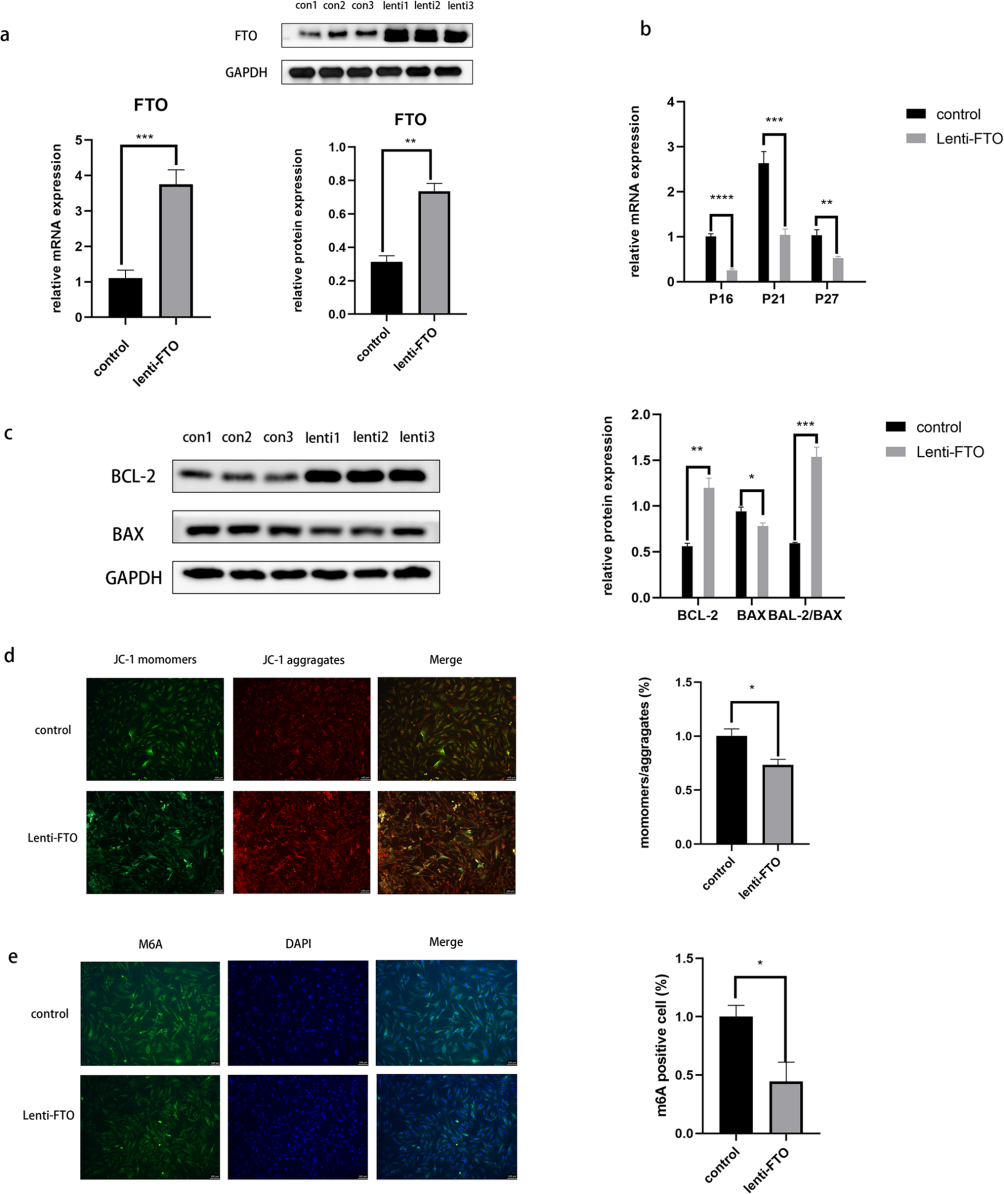
Overexpression of FTO inhibits granulosa cell senescence. (**a**) FTO expression level after transfecting with lentivirus-FTO; (**b**) qPCR results revealed the mRNA levels of P16, P21, and P27 after FTO overexpression; (**c**) Western blot results indicated the protein levels of Bax and Bcl‐2 after FTO overexpression; (**d**) intracellular MMP was measured using JC-1 staining in the control and FTO overexpression groups; (**e**) Immunofluorescence results showed RNA m6A modification level after FTO overexpression. *P < 0.05; **P < 0.01; ***P < 0.001; ****P < 0.0001. FTO, fat mass and obesity-associated protein; qPCR, quantitative polymerase chain reaction; MMP, mitochondrial membrane potential

A lentivirus was used to infect KGN cells to overexpress FTO to further elucidate the role of FTO (Fig. 3a). RT-qPCR analysis revealed that increased FTO expression decreased the transcriptional levels of P16, P21, and P27 (Fig. 3b). Additionally, FTO overexpression resulted in a reduced expression of Bax and increased levels of Bcl-2 and BCL-2/BAX ratio (Fig. 3c). Furthermore, FTO overexpression led to a decreased proportion of cells positive for an increased MMP (Fig. 3d) and decreased RNA m6A modification levels (Fig. 3e), indicating that FTO overexpression impedes the process of granulosa cell senescence.

## MMP2 Participates in the Granulosa Cell Aging Process

**Fig. 4:**
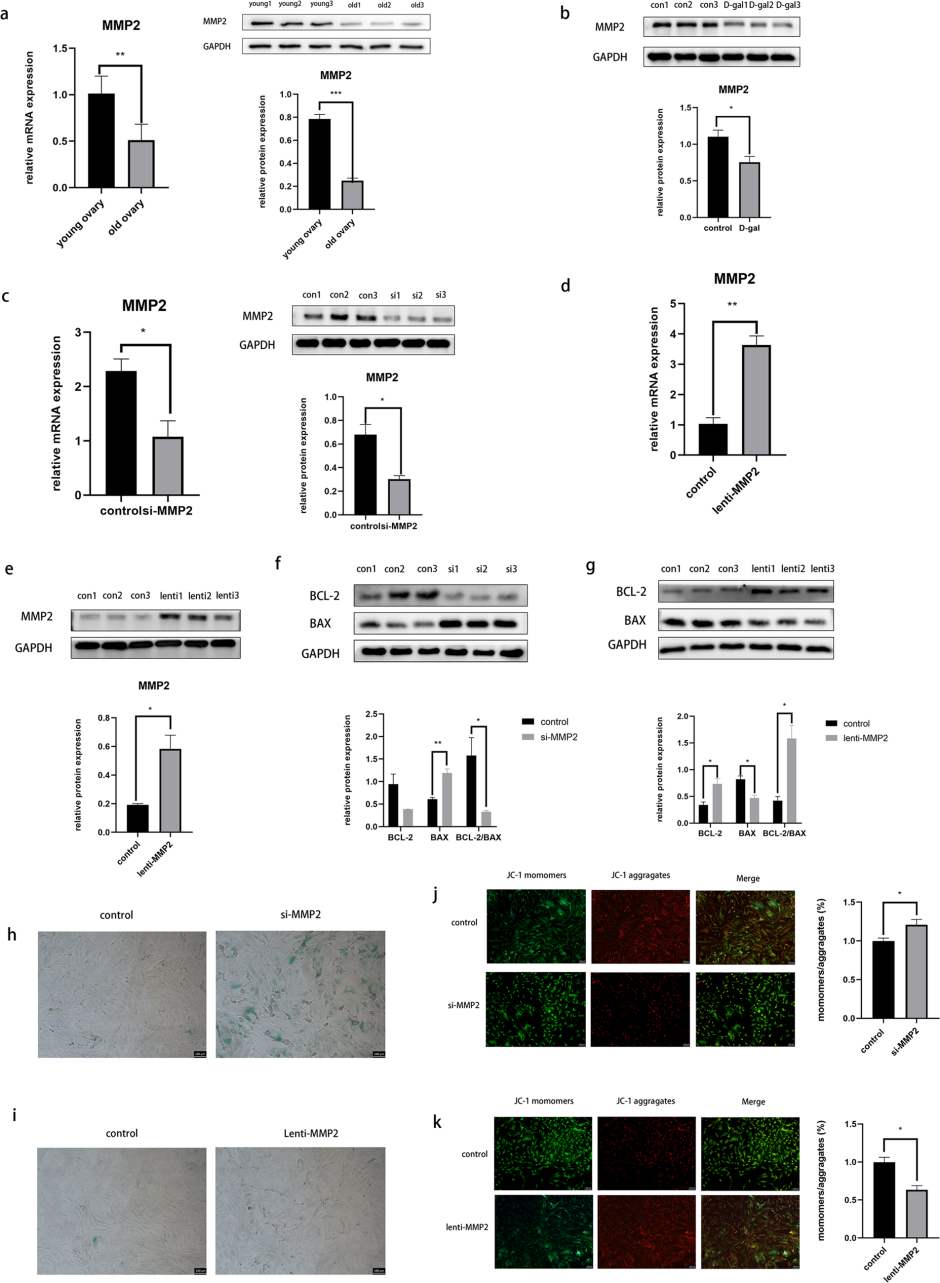
MMP2 participates in the granulosa cell aging process. (**a**,**b**) qPCR and Western blot results showed MMP2 protein expression in aged mice’s ovaries and in the D-( +)-Galactose treatment senescent cells; (**c**) MMP2 expression level after transfecting with si-MMP2; (**d**, **e**) MMP2 expression levels after transfecting with lentivirus-MMP2; (**f**, **g**) Western blot results indicated the protein levels of Bax and Bcl‐2 after MMP2 knockdown and overexpression; (**h**, **i**) the β-galactosidase staining detected senescent cells in the MMP2 knockdown and overexpression groups; (**j**, **k**) intracellular MMP was measured using JC-1 staining in the control and MMP2 downregulated and overexpression groups. *P < 0.05; **P < 0.01; ***P < 0.001. FTO, fat mass and obesity-associated protein; MMP2, matrix metalloproteinase 2

Decreased MMP2 expression was observed in aged mouse ovaries and senescent granulosa cells (Figs. 4a and b). siMMP2 was transfected into granulosa cells to investigate the role of MMP2 in granulosa cell aging, the qPCR and Western blot results confirmed the success of this experiment (Fig. 4c). MMP2 interference reduced Bcl-2 protein levels and increased Bax levels (Fig. 4f). Subsequent β-galactosidase-stain revealed higher positive stained cells (Fig. 4h) and MMP staining revealed reduced MMP levels in the si-MMP2 group of KGN cells (Fig. 4j), indicating that MMP2 knockdown promoted granulosa cell senescence. We explored the effects of MMP2 overexpression on KGN cell senescence (Fig. 4d and e). MMP2 overexpression in KGN cell lines resulted in decreased BAX protein levels, increased Bcl-2 levels (Fig. 4g), and increased MMP levels (Fig. 4k), suggesting that MMP2 overexpression delayed the senescence of KGN cells. There was no significant difference in the proportion of β-galactosidase-stained cells between the mmp2 overexpression groups and the control group (Fig. 4i). The overexpression of the MMP2 gene is known to promote cell proliferation. Additionally, since the cells in the control group were also in a proliferative state without any intervention, the lack of significant difference between the two groups can be attributed to this factor.

## FTO Regulates MMP2 Expression in an m6A-YTHDF2-Dependent Manner

**Fig. 5:**
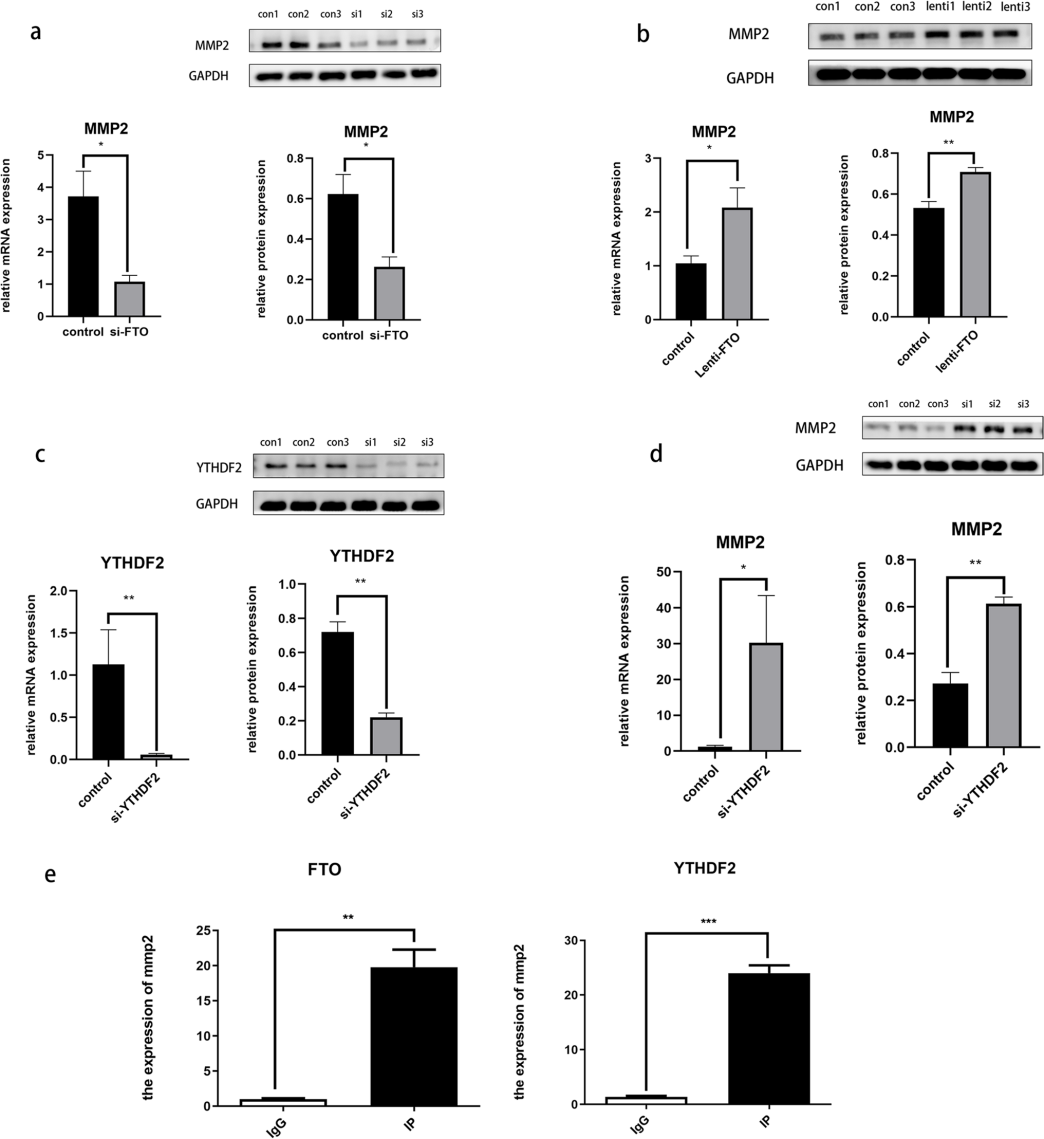
FTO regulates MMP2 expression in an m6A-YTHDF2-dependent manner. (**a**, **b**) MMP2 mRNA and protein levels were analyzed using q-PCR and Western blot after FTO knockdown and overexpression; (**c**) q-PCR and Western blot results indicated YTHFD2 was downregulated by si-YTHDF2; (**d**) MMP2 mRNA and protein levels were analyzed using q-PCR and Western blot after YTHDF2 knockdown; (**e**) RIP results indicate the interaction between MMP2 mRNA transcripts and FTO and YTHDF2 proteins. *P < 0.05; **P < 0.01; ***P < 0.001. FTO, fat mass and obesity-associated protein; MMP2, matrix metalloproteinase 2; qPCR, quantitative polymerase chain reaction; RIP, RNA immunoprecipitation

Since both FTO and MMP2 are involved in cellular senescence, the question arises as to whether FTO influences MMP2 expression via RNA m6A methylation. MMP2 mRNA and protein levels were measured in KGN cells transfected with si-FTO or lentivirus-FTO, respectively. The results demonstrated that FTO knockdown led to a significant decrease in MMP2 expression compared with that in control cells (Fig. 5a). However, the overexpression of FTO had the opposite effect on MMP2 levels (Fig. 5b), indicating that FTO mediates MMP2 expression through a demethylation-dependent pathway. Subsequent investigations focused on the effects of FTO on the MMP2 protein levels. Previous studies have shown that YTHDF2 selectively recognizes m6A modifications and mediates the degradation of m6A-containing mRNA [13], suggesting a potential regulatory role for YTHDF2 in MMP2 expression. We hypothesized that m6A-containing MMP2 mRNAs are recognized and bound by YTHDF2, leading to their degradation. To test this hypothesis, si-YTHDF2 was transfected into KGN cells (Fig. 5c), upregulating MMP2 expression (Fig. 5d). Subsequent RIP assays revealed that the mRNA of MMP2 could bind to both FTO and YTHDF2 proteins (Fig. 5e), indicating that FTO and YTHDF2 are directly involved in the post-transcriptional regulation of MMP2.

## The ERK Signaling Pathway Regulates Granulosa Cell Senescence as a Downstream of MMP2

**Fig. 6:**
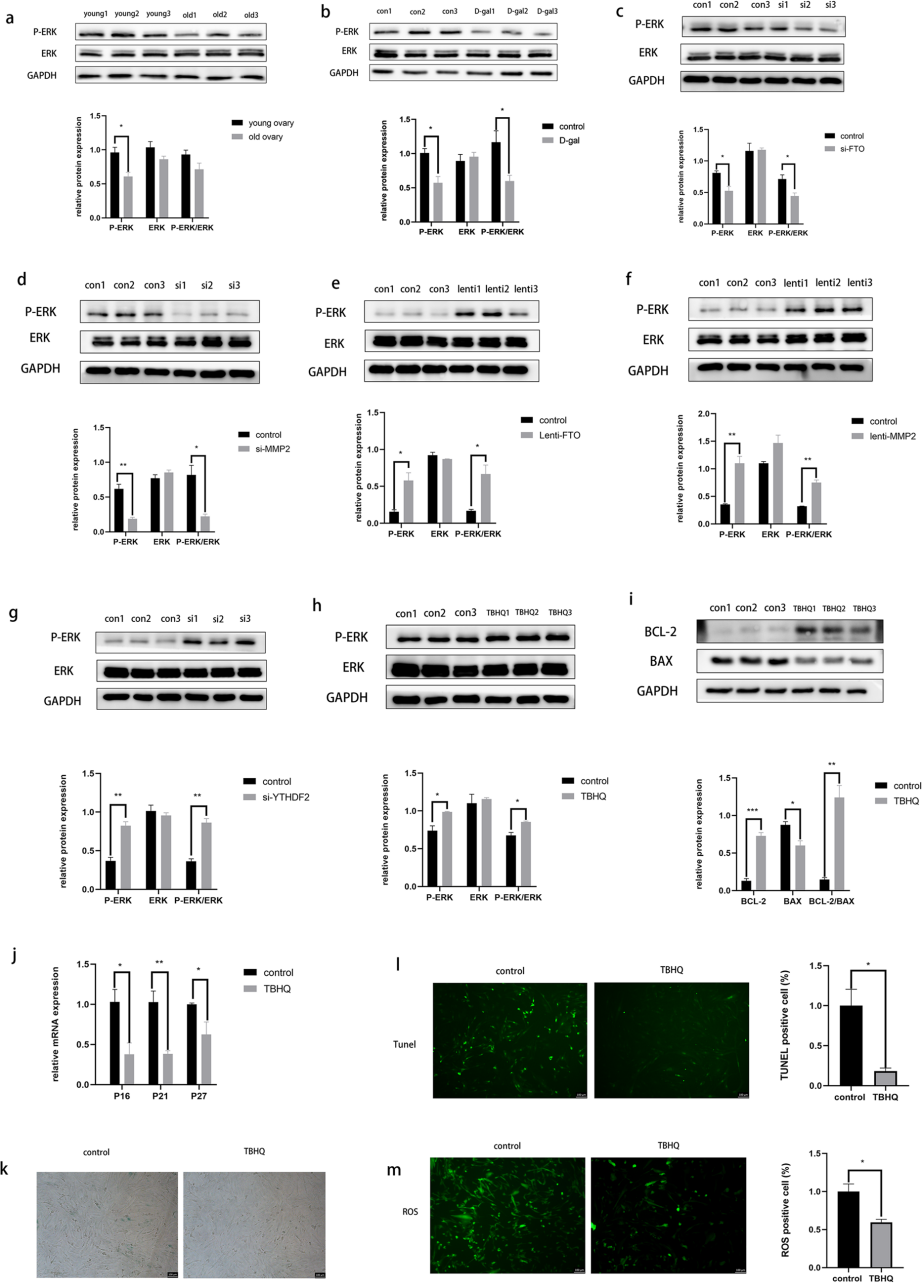
The ERK signaling pathway regulates granulosa cell senescence as a downstream of MMP2. The ERK and p‐ERK protein levels were analyzed using Western blot (**a**) in aged mice’s ovaries, (**b**) after D-( +)-Galactose treatments in KGN cells, (**c**, **d**) after FTO and MMP2 knockdown, (**e**, **f**) after FTO and MMP2 overexpression, (**g**) after YTHDF2 knockdown, (**h**) and after TBHQ treatment; (**i**) Western blot results indicated the protein levels of Bax and Bcl‐2 after TBHQ treatment; (**j**) qPCR results revealed the mRNA levels of P16, P21, and P27 after TBHQ treatment; (**k**) the β-galactosidase staining detected senescent cells in the control and TBHQ treatment groups; (**l**) The TUNEL assay detected senescent cells in the control and TBHQ groups. Green fluorescence represents senescent cells; (**m**) the relative levels of reactive oxygen species in the control and TBHQ groups. *P < 0.05; **P < 0.01; ***P < 0.001. ERK, extracellular signal-regulated kinase; MMP2, matrix metalloproteinase; FTO, fat mass and obesity-associated protein; TBHQ, tert-butylhydroquinone

ERK signaling pathway plays an important role in cell growth, proliferation, differentiation, senescence, and apoptosis, and the relationship between the ERK pathway and granulosa cell senescence was also investigated. Western blot assays demonstrated a significant reduction in the activation of the ERK pathway in the ovaries of aged mice (Fig. 6a) and D-( +)-galactose-induced senescent cells (Fig. 6b). In this study, we aimed to explore the connection between the ERK signaling pathway and FTO/YTHDF2/MMP2 in an m6A-dependent manner. Further experiments revealed that the knockdown of FTO and MMP2 in KGN cells weakened the activation of the ERK signaling pathway (Fig. 6c and d). However, FTO and MMP2 overexpression (Fig. 6e and f) and YTHDF2 knockdown (Fig. 6g) significantly activated the ERK pathway. This indicated that the ERK signaling pathway is a downstream target of MMP2, which regulates granulosa cell senescence. Western blot showed that tert-butylhydroquinone (TBHQ), an activator of the ERK pathway (Fig. 6h), increased Bcl-2 expression and decreased Bax expression (Fig. 6i). RT-qPCR analysis revealed that activation ERK pathway decreased the transcriptional levels of P16, P21, and P27 (Fig. 6j). The proportion of β-galactosidase-stained cells was significantly lower in the TBHQ treated KGN cells compared with control cells (Fig. 6k). Additionally, TUNEL results showed a decreased proportion of TUNEL-positive cells (Fig. 6l) and reduced intracellular ROS levels (Fig. 6m) after TBHQ treatment. These findings suggest that activating the ERK pathway can effectively delay senescence in KGN cells.

## Discussion

The ovarian follicle is a fundamental functional unit consisting of granulosa cells and oocytes. As the ovaries age, the functions of the granulosa cells and oocytes naturally decline [23]. Granulosa cells surround the oocyte in a multilayered structure and communicate with it either directly through the zona pellucida or indirectly through paracrine signaling. They play a crucial role in regulating the growth and development of the oocyte [2]. Additionally, granulosa cells in the follicle provide nutrients to the oocyte, maintain it in an arrested state, and trigger meiosis when necessary [3, 4]. Aging leads to structural degeneration in DNA, proteins, and lipids in granulosa cells, which hinders the developmental potential of oocytes [24]. Studies have shown that removing oocytes from the aging follicular microenvironment and maturing them in vitro can partially mitigate the effects of age-related defects [25]. Therefore, any factor that triggers senescence in granulosa cells can affect follicular development, resulting in decreased ovulation rate and reduced fertility. Consequently, investigating the potential pathways and mechanisms that lead to granulosa cell senescence is essential for delaying ovarian aging.

Our study confirmed the involvement of FTO in the aging of granulosa cells by manipulating its expression levels. Previous research has shown a decreased expression of the m6A demethylase FTO in aging ovaries [22]. This reduced expression in aging ovaries and cells may be due to two main factors. First, the FTO-dependent RNA m6A modification regulates cell proliferation, aging, apoptosis, and other cellular processes. Studies have shown that the growth and metabolism of FTO knockout models are reduced in failing mammalian hearts and hypoxic cardiomyocytes [26]. Additionally, FTO expression is upregulated in metabolic diseases such as cervical cancer, breast cancer, and diabetes [27–29]. However, studies have found an increased FTO expression in the peripheral blood mononuclear cells of older adults, suggesting tissue-specific regulation [30]. Second, FTO overexpression has been linked to reduced fibrosis and enhanced angiogenesis [26], potentially explaining the higher risk of ovarian interstitial fibrosis, an early sign of ovarian aging, in older women.

Decreased MMP2 levels have been observed in the ovaries and aged mice granulosa cells, suggesting its involvement in the aging process of granulosa cells. Knockdown and overexpression experiments confirmed these results. Further experiments showed that MMP2 mRNA could bind to FTO and YTHDF2, indicating a direct regulatory process. YTHDF2 regulates MMP2 degradation by recognizing FTO-mediated m6A modifications in MMP2. Similar mechanisms have been observed in melanoma and rectal cancer studies, where FTO downregulation led to increased m6A methylation levels in key genes, resulting in enhanced RNA degradation by YTHDF2 [31, 32]. However, a study on reproductive toxicity in mouse Leydig cells caused by bisphenol F revealed a different role for YTHDF2, which involved enhancing the stability of the target gene nuclear factor erythroid 2-related factor 2 mRNA rather than degrading it [33]. This discrepancy may be due to the different nature of the conditions studied, with endogenous factors in melanoma, colon cancer, and ovarian aging, and an exogenous poison, such as bisphenol F. However, further research is needed to fully understand these implications.

*MMP2*, a target gene regulated by FTO/YTHDF2, is crucial for the cyclic remodeling of ovarian tissue during ovulation cycles [34]. Our study suggests that as ovarian aging progresses, MMP2 expression decreases, disrupting tissue remodeling homeostasis and leading to ovarian fibrosis. Additionally, we discovered that MMP2, an upstream regulator of the ERK pathway, affects granulosa cell aging by modulating ERK pathway activation. Previous research has shown that ERK pathway activation typically drives immortal cell transformation into cancer cells [35]. However, in some cases, aberrant ERK activation can promote senescence and cell death in primary cells [36]. Notably, our findings demonstrated that ERK activation can inhibit cell aging, as the normal ERK pathway promotes cell growth and proliferation. This indicates that, during ovarian aging, the ERK signaling pathway in granulosa cells remains normally activated and continues to influence cell aging, although with a gradually diminishing proliferative effect.

However, this study had some limitations. This experimental study was limited to mice and cells, and it remains uncertain whether the aging pathways discussed apply to human ovaries and granulosa cells. Additionally, this study focused solely on activating the ERK pathway without investigating the use of inhibitors. However, further research is required to validate these findings.

## Conclusion

This study investigated the differential levels of RNA m6A modification and protein expression in the ovaries of young and aged mice. The observed differences in expression were replicated in a granulosa cell aging model. Subsequent experiments involved KGN Knockdown and the overexpression of FTO, YTHDF2, and MMP2 to examine their effects on cell aging. Molecular pathway analyses were conducted to elucidate the aging process, which revealed the presence of epigenetic RNAs in granulosa cells. Specifically, we identified an aging pathway associated with m6A modification, in which reduced FTO expression in senescent granulosa cells led to an increased methylation modification of MMP2. This modification facilitates the recognition and degradation of MMP2 by YTHDF2, resulting in decreased MMP2 expression, diminished ERK pathway activation, and ultimately accelerated aging.

## Fundings

This research was supported by the National Natural Science Foundation of China (No.81771582) and the Hubei Provincial Natural Science Foundation of China (No.2023AFB698).

## Data Availability

The datasets generated during and/or analysed during the current study are available from the correspondingauthor on reasonable request.
